# Never-Told Tales

**Published:** 1910-12

**Authors:** 


**Never-Told Tales.** By **William J. Robinson,** M.D., Editor of the American Journal of Urology, etc. Third edition. 12 mo, pp. 155. New York: The Altrurians, Publishers. (Cloth, $1.00.)

Never-Told Tales are a series of short stories written in Dr. Robinson's well-known style, on subjects not usually discussed in polite society. As three editions have been called for during the past two years there must be a demand for the book. The author thinks that if these tales were put into the hands of every man and woman about to marry, and into the hands of every father and mother who have grown children, much misery would be prevented, and much good accomplished. J. A. R.

